# Oxic methanogenesis is only a minor source of lake-wide diffusive CH_4_ emissions from lakes

**DOI:** 10.1038/s41467-021-21215-2

**Published:** 2021-02-22

**Authors:** F. Peeters, H. Hofmann

**Affiliations:** Environmental Physics Group, Limnological Institute, University of Konstanz, Konstanz, Germany

**Keywords:** Climate sciences, Environmental sciences, Limnology, Water resources

**Arising from** M. Günthel et al. *Nature Communications* 10.1038/s41467-019-13320-0 (2019)

Methane emissions from lakes are a major natural source in the global budget of atmospheric methane. A large fraction of these emissions result from diffusive CH_4_ emissions, i.e., the diffusive transport of oversaturated methane from the surface waters to the atmosphere. CH_4_ is typically produced in anoxic sediments and oxidized in oxic waters^1^ but can also be produced in oxic waters^2,3^. Schmidt and Conrad^4^ suggested that the oversaturation of CH_4_ in surface waters of lakes results from two processes: CH_4_ release from littoral sediments in combination with horizontal transport to the open water and in situ net production of CH_4_ in oxic surface water. The relative importance of the two processes for diffusive emissions of CH_4_ from lakes is the focus of this discussion.

Günthel et al.^5^ claim that oxic methanogenesis contributes the main fraction, i.e., up to 100%, of the CH_4_ emitted from lakes with surface area >1 km^2^. Their conclusion is based on their Fig. 4^5^, which combines results from re-analyses of Donis et al.^6^ and DelSontro et al.^7^ and from CH_4_ mass balances for Lake Stechlin. We demonstrate below that the analyses of Günthel et al.^5^ contain several errors. Without these errors, the data do not support their main conclusion but suggest that CH_4_ fluxes from littoral zones are the dominant source of diffusive CH_4_ emissions from all lakes independent of their size. The latter is consistent with the analysis of DelSontro et al.^7^ and the conclusions of Encinas Fernandez et al.^8^ and Peeters et al.^9^.

In the following, we first explain our approach estimating the contribution of net oxic methane production to the total diffusive CH_4_ emissions, NOMC (net oxic methane production contribution). We then clarify several errors and inconsistencies in the analyses of Günthel et al.^5^ and summarize the corrected results in Fig. 1. These new results on NOMC are discussed in relation to other studies commenting also on the limitations of mass balance and of other approaches to estimate NOMC.

**Fig. 1:**
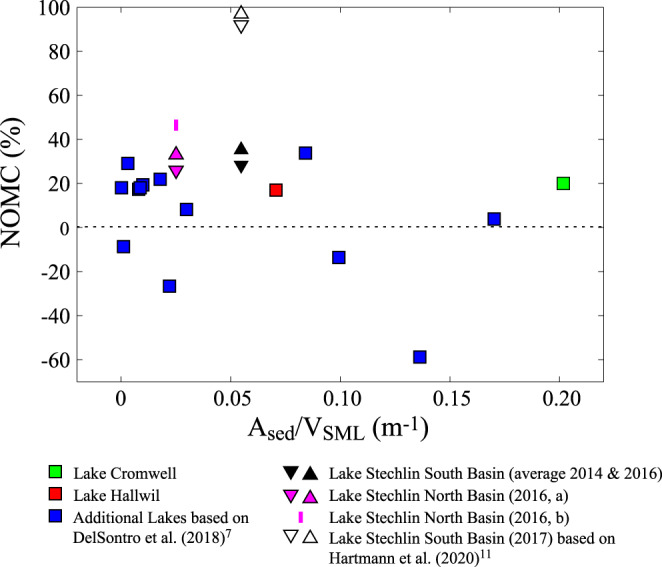
The contribution of net oxic methane production to the diffusive CH_4_ emission from lakes. The contribution of net oxic methane production to the diffusive CH_4_ emission, NOMC, was calculated in the different lakes from the surface CH_4_ flux, *F*_surf_, and the methane flux from the sediments, *F*_sed_, obtained from different data sources: Lake Hallwil (Supplementary Table 1): *F*_surf_ from the “Hallwil relationship” that is based on the chamber measurements in Lake Hallwil^6^. *F*_sed_, from the CH_4_ pore water concentrations in the sediment core collected at 3 m water depth (*F*_sed_ = 2.8 mmol m^−2^ day^−^^1^, Supplementary Table 1). Lake Stechlin (Supplementary Table 3): Lower and upper limits of *F*_sed_ (*F*_sed_ = 1.8 mmol m^−2^ day^−1^ and *F*_sed_ = 2.0 mmol m^−2^ day^−1^) from the re-evaluation of the mesocosm experiments (Supplementary Table 2) providing upper and lower limit of NOMC, respectively (Supplementary Table 3). South Basin (average 2014, 2016): *F*_surf_ from the “Stechlin relationship”; North Basin (2016, a): *F*_surf_ from chamber measurements; North Basin (2016, b) *F*_surf_ from chamber measurements combined with the “Stechlin relationship” for the 20 June; Lake Stechlin South Basin (2017) (Supplementary Table 4): *F*_sed_ derived from CH_4_ pore water measured in a single sediment core by ref. ^11^, considering the CH_4_ gradient in the top 2 cm and at 5 cm depth (*F*_sed_ = 0.08 mmol m^−2^ day^−1^ and *F*_sed_ = 0.26 mmol m^−2^ day^−1^) providing upper and lower limit of NOMC, respectively; *F*_surf_ from specific wind model of ref. ^11^; Lake Cromwell: Data from ref. ^5^. Additional Lakes (Supplementary Table 5): based on the analysis of ref. ^7^ (see Supplementary Note 4). The ratio between the area of the sediment *A*_sed_ and the volume *V*_SML_ in the surface mixed layer SML, *A*_sed_*/V*_SML_, was estimated assuming a slope angle of 5° for the lake bed (Supplementary Note 4). The sensitivity to the slope angle is illustrated in Supplementary Fig. 5 assuming a slope angle of 3°.

## Determination of the contribution of oxic methanogenesis to diffusive CH_4_ emissions

Net production of methane in oxic waters (NOM) in the surface mixed layer (SML) is estimated as the difference between the total diffusive CH_4_ emissions from the lake surface, *F*_surf,tot_, and the total flux from the sediments in the SML, *F*_sed,tot_, i.e., NOM = *F*_surf,tot_ − *F*_sed,tot_. This procedure neglects processes contributing to the mass balance, e.g., vertical transport of CH_4_ into the SML, but allows for a consistent comparison of the observations from Lake Hallwil and Lake Stechlin with the results derived from data of DelSontro et al.^7^. Note that the neglected processes are typically small^5^ or uncertain (e.g., estimates of turbulent diffusivities in the thermocline have large uncertainty; the amount and dissolution of microbubbles were not measured in the studies considered by ref. ^5^) and are sources of CH_4_ in Lake Hallwil and Lake Stechlin^5^. Our estimates are therefore upper limits of NOM. The contribution of NOM to overall diffusive CH_4_ emission is defined as: NOMC = NOM/*F*_surf,tot_. The advantage of using NOM instead of gross production of CH_4_ is explained in Supplementary Note 1.

## Re-evaluation of the analysis of Günthel et al.^5^: data of Donis et al.^6^ from Lake Hallwil

In Lake Hallwil, the contribution of oxic methanogenesis to overall diffusive CH_4_ emissions has been estimated to be 90%^6^ or 63–83%^5^, but we show here that NOMC ~ 17%.

In the mass balance of the SML extending from 0 to 5 m water depth^5,6^, Günthel et al.^5^ used an average sediment flux of *F*_sed_ = 1.75 mmol m^−^^2^ day^−1^, averaging flux estimates of Donis et al.^6^ from two sediment cores, one collected at 3 m and the other at 7 m water depth. The δ^13^C of the CH_4_ in the pore water of these two cores differ substantially^6^, indicating differences in production and oxidation of CH_4_ between the sediments in the SML and at 7 m water depth. The estimate of *F*_sed_ in the SML should therefore be based on the core collected at 3 m water depth. Using the approach of Donis et al.^6^, the correct *F*_sed_ derived from the data of this core is *F*_sed_ = 2.8 mmol m^−^^2^ day^−1^ (Peeters et al.^9^, see Supplementary Note 2.1 for details).

Günthel et al.^5^ and Donis et al.^6^ apparently have erroneously used gas transfer coefficients instead of proper CH_4_ fluxes to calculate emissions. This conclusion is demonstrated by the perfect agreement between the values published erroneously as CH_4_ fluxes, *F*_surf_, by Günthel et al.^5^ and the values of the gas transfer coefficients of CH_4_ at 20 °C, *k*_CH4_, calculated by us (Table 1). The values published by Donis et al.^6^ as CH_4_ fluxes are very similar to these *k*_CH4_ and therefore also do not represent CH_4_ fluxes but gas transfer coefficients (for details, see Supplementary Note 2.2).

**Table 1:** Average CH_4_ surface fluxes from Lake Hallwil and gas transfer velocities.

Model			*F*_surf,G_^5^ (mmol m^−2^ day^−1^)	*k*_600_ (m day^−1^)	*k*_CH4_ (20 °C) (m day^−1^)	*F*_surf_ (20 °C) (mmol m^−2^ day^−1^)
Flux chamber^6^			0.6	(1.8)……**0.6**	(1.8)……**0.6**	(0.6)……**0.18**
Hallwil relationship^6^	*k*_600,m_ = 2 * *U*_10_	(cm h^−1^)	0.8	0.8	0.8	0.24
MacIntyre et al.^10^						
Positive buoyancy flux	*k*_600,m_ = 1.74 * *U*_10_ − 0.15	(cm h^−1^)	0.7	0.7	0.7	0.21
Negative buoyancy flux	*k*_600,m_ = 2.04 * *U*_10_ + 2	(cm h^−1^)	1.3	1.3	1.3	0.39
Combined buoyancy flux	*k*_600,m_ = 2.25 * *U*_10_ + 0.16	(cm h^−1^)	1.0	1.0	0.9	0.27
Vachon and Prairie^17^	*k*_600,m_ = 1.48 * *U*_10_ + 2.51 + 0.39 * *U*_10_ * log_10_(*A*_surf_)	(cm h^−1^)	1.4	1.4	1.3	0.39

Average CH_4_ surface fluxes estimated by Günthel et al.^5^, *F*_surf,G_, are compared to gas transfer velocities and correct CH_4_ surface fluxes at 20 °C. Donis et al.^6^ published the same value as Günthel et al.^5^ for the flux chamber measurements and 0.8 mmol m^−2^ day^−1^ for the MacIntyre model with positive buoyancy flux. *k*_600_ is the gas transfer velocity for CO_2_ at 20 °C, *k*_CH4_ (20 °C) the gas transfer velocity of CH_4_ at 20 °C, and *F*_surf_ (20 °C) the surface flux of CH_4_ at 20 °C calculated by us. *F*_surf_ (20 °C) is calculated from *k*_CH4_ (20 °C) and using for the surface concentration 0.3 mmol m^−3^ in Lake Hallwil^5,6^. *U*_10_ is the wind speed 10 m above the lake surface and *A*_surf_ is the surface area of the lake. Details on the calculations are provided in Supplementary Note 2.

Note that Günthel et al.^5^ used wind speed data from 15 April to 28 July but not from August. We applied here the same to allow direct comparison. The average *U*_10_ of these data is 1.69 m s^−1^. In case of flux chambers, the numbers in brackets use the values of Günthel et al.^5^ and the surface concentration 0.3 mmol m^−3^ in Lake Hallwil^5,6^. Bold numbers assume that the flux chamber data of Donis et al.^6^ used by Günthel et al.^5^ represent *k*_600_ and not *F*_surf_, as is supported in the main text and in Supplementary Note 2.

The gas transfer coefficient of CH_4_ must be multiplied by the difference between the surface concentration (0.3 mmol m^−^^3^, ref. ^6^) and the atmospheric equilibrium concentration of CH_4_ (CH_4,equ_ = 0.003 mmol m^−3^ at 20 °C^9^), i.e. by ~0.3 mmol m^−3^, to obtain *F*_surf_. *F*_surf_ is therefore ~3.3 times smaller than the values of the gas transfer coefficients erroneously taken by Günthel et al.^5^ and Donis et al.^6^ as CH_4_ fluxes (Table 1 and details in Supplementary Note 2.2).

Donis et al.^6^ and Günthel et al.^5^ used values obtained from measurements with floating chambers to calculate emissions, but these values claimed to represent *F*_surf_ appear to be in fact values for transfer coefficients, suggesting the same mistake as in the case of the wind models. Donis et al.^6^ stated: “Average flux (April–August 2016) is equal to 0.8 ± 0.2 mmol m^−2^ d^−1^ from MacIntyre relationship for positive buoyancy and to 0.6 ± 0.3 mmol m^−2^ d^−1^ from chamber measurements. The latter, not significantly different from the wind-based relationship, was used for the mass balance”. Günthel et al.^5^, co-authored by D. Donis, claim that the “MacIntyre relationship for positive buoyancy”^10^ provides an average value of 0.7 for *F*_surf_, but in fact 0.7 is the average value for *k*_CH4_ in unit m day^−1^ (0.7 m d^−1^, see Table 1) and *F*_surf_ for this model is 3.3 times smaller (0.21 mmol m^−2^ d^−1^, see Table 1). The value by Donis et al.^6^ for the MacIntyre relationship^10^ is even slightly larger than 0.7 and therefore clearly incompatible with *F*_surf_ but is rather a gas transfer coefficient as is obvious in the case of Günthel et al.^5^. The good agreement between the value for the gas transfer coefficient obtained from the MacIntyre model for positive buoyancy flux^10^ and the values from the chamber measurements suggests that the values from the chamber measurements are not gas fluxes but also gas transfer coefficients (see Supplementary Note 2.2 for more details).

Donis et al.^6^ derived from their chamber measurements the wind-based model “Hallwil relationship” specifically for Lake Hallwil. The establishment of this Hallwil relationship required that Donis et al.^6^ calculated gas transfer coefficients from their chamber measurements. In their Supplementary Fig. 4, Donis et al.^6^ show that the values from their chamber measurements agree well with those from the Hallwil relationship (Supplementary Fig. 2 and Supplementary Note 2.2). Note, however, that the values for the Hallwil relationship are in fact gas transfer coefficients and not *F*_surf_, supporting that also the values from the chamber measurements represent gas transfer coefficients and not *F*_surf_ (Supplementary Fig. 2 and Supplementary Note 2.2 for more details). This conclusion implies that the values from the chamber measurements by Donis et al.^6^ must be multiplied by ~0.3 mmol m^−3^ to give proper CH_4_ fluxes, which are then ~3.3 times smaller than the CH_4_ fluxes used in the mass balances of refs. ^5,6^.

Because there are only four chamber measurements available for 2016 and one of them was exceptionally low (see ref. ^6^ and Supplementary Note 2.2), the Hallwil relationship is considered here to provide the most reliable estimate of the average *k*_600_ in Lake Hallwil and therefore applied to calculate the average surface CH_4_ flux for April to August 2016, i.e., *F*_surf_ = 0.24 mmol m^−2^ d^−1^ (see Table 1 and Supplementary Note 2.2). The reliability of the Hallwil relationship was confirmed by Günthel et al.^5^ and by Hartmann et al.^11^ comparing different estimates of surface fluxes in the South Basin of Lake Stechlin.

With *F*_sed_ = 2.8 mmol m^2^ day^−1^ and *F*_surf_ = 0.24 mmol m^2^ day^−1^, NOM = 416 mol day^−1^ and the contribution of NOM to total emissions is NOMC = 17% (Supplementary Table 1 in Supplementary Note 2.3 includes also additional estimates of NOMC). The low value of NOMC suggests that most of CH_4_ in the SML originates from the sediments, which is consistent with the δ^13^C isotopic composition of CH_4_ in Lake Hallwil^9^. The uppermost CH_4_ in the sediment core from the SML is characterized by δ^13^C about –59‰, which corresponds very closely to the δ^13^C of the CH_4_ in the open water of the SML ranging from −62‰ to −58‰ (Figs. 4 and 5 both in ref. ^6^). Thus the δ^13^C values suggest that the CH_4_ from the uppermost pore water in the sediment of the SML is the source of the CH_4_ in the open water and do not indicate a reduction of the δ^13^C expected in case of substantial CH_4_ production.

## Re-evaluation of the analysis of Günthel et al.^5^: data from Lake Stechlin

Günthel et al.^5^ underestimated the sediment flux and overestimated the emissions in Lake Stechlin and thus overestimated NOM (see below). We therefore re-evaluated the mass balances from Lake Stechlin to provide NOMC that are presented in Fig. 1 (see also Supplementary Table 3 in Supplementary Note 3.2). Oxic methanogenesis in Lake Stechlin was determined by Günthel et al.^5^ using the same mass balance approach as in Lake Hallwil, but the sediment flux was estimated from a mesocosm experiment that involved two mesocosms. CH_4_ surface fluxes from the two mesocosms were utilized to calculate CH_4_ production within the mesocosms. Assuming that CH_4_ production in the SML of the lake is the same as in the mesoscosm, *F*_sed_ was determined by closing the mass balance of the SML in the lake. However, *F*_surf_ from the mesocosms was overestimated because the gas transfer coefficient *k*_600_ (transfer coefficient of CO_2_ at 20 °C) determined for the open water of the lake was also used for the mesocosms^5^. The turbulence in the mesocosm is substantially lower than in the open water, i.e., in the uppermost 1 m of measurements the energy dissipation *ε* in the lake is 5–10 times larger than in the mesocosm (for details, see Supplementary Note 3.1; values on energy dissipation *ε* are from the data source of Supplementary Fig. 8 in Günthel et al.^5^). Because *k*_600_ ~ *ε*^¼ ^^12–14^, the difference in energy dissipation between lake and mesocosm suggest that *k*_600_ in the mesocosm should be scaled by 5^−^^¼^ to 10^−¼^ and is therefore only 67 or 56%, respectively, of the *k*_600_ in the lake (see Supplementary Note 3.1). Hence, *F*_surf_ in the mesocosm is only 67 or 56% of the value used by Günthel et al.^5^ and the lower and upper bounds of the sediment flux become *F*_sed_ = 1.8 and 2.0 mmol m^−2^ day^−1^, respectively (Supplementary Table 2 and Supplementary Note 3.1) and thus are substantially larger than *F*_sed_ = 1.4 mmol m^−2^ day^−1^ used by Günthel et al.^5^.

The sediment flux derived from the mesocosm experiments conducted in the South Basin of Lake Stechlin in 2014 was also used for the stratified periods in 2016 and 2018 and in both basins of Lake Stechlin^5^. Hence the underestimation of the sediment flux in 2014 resulted in an overestimation of net production of CH_4_ in all results of Günthel et al.^5^.

NOMC calculated from the mass balance in the South and North Basin of Lake Stechlin, using the sediment fluxes corrected for the difference in turbulence between lake and mesocosms, are lower than 40% and agree well between 2014 and 2016 and between the basins (Supplementary Table 3 and detailed analysis in Supplementary Note 3.2).

Hartmann et al.^11^ collected in 2017 one sediment core from the SML in the South Basin of Lake Stechlin and provided another wind model for *k*_600_. Re-analysis of the CH_4_ pore water of the sediment core provides a sediment flux into the water of 0.08–0.26 mmol m^−2^ day^−1^ (Supplementary Note 3.3) This flux is exceptionally low and incompatible with the sediment flux derived from the mesocosm experiments for the same basin, suggesting that the flux estimate based on a single sediment core is not representative for the average *F*_sed_ in the SML. NOMC derived from this sediment flux and the model of *k*_600_ of ref. ^11^ is exceptionally high (Fig. 1, Supplementary Table 4, and Supplementary Note 3.3).

However, in addition to our re-analysis of the data of ref. ^5^ for Lake Stechlin, there is further evidence that NOMC is typically not very large in Lake Stechlin. According to Fig. 3 in Günthel et al.^5^, oxic CH_4_ production was small in 2018 and even negative in the South Basin, implying NOMC < 0. Apparently, net oxidation instead of net production of CH_4_ was the dominant process in the South Basin in 2018.

Furthermore, in the central mesocosm (central reservoir) in Lake Stechlin, which was disconnected from the littoral CH_4_ source for a very long time period, CH_4_ concentrations were very low and close to atmospheric saturation^5^. Emissions from this mesocosm were therefore very small^5^ showing no indication of significant in situ production of CH_4_. The mesocosms used for estimating oxic methanogenesis in Lake Stechlin were measured within 10 days after their filling and possibly had not reached steady state.

## Re-evaluation of the analysis of Günthel et al.^5^: data of DelSontro et al.^7^ from additional lakes

Günthel et al.^5^ re-analyzed data from 7 lakes originally investigated by DelSontro et al.^7^ and claim that in these lakes oxic methane production contributes between 82 and 100% of the total CH_4_ emissions. However, these values are incompatible with the average net production of 25% stated by DelSontro et al.^7^ for their systems with positive net production. Furthermore, according to DelSontro et al.^7^ net production was negative in 30% of their lakes suggesting that in these lakes 100% of the emitted CH_4_ was provided by CH_4_ fluxes from the littoral zone. One of these lakes with negative net production was Lake Champlain^7^, but Günthel et al.^5^ claim that in this lake 100% of the emissions originate from oxic methane production.

It is unclear how Günthel et al.^5^ performed the analysis of the data of DelSontro et al.^7^ (see Supplementary Note 4.1 for details). We therefore determined NOMC for all lakes studied by DelSontro et al.^7^ (Figs. 1, S4, and S5; Tables 2 and S5, and details in Supplementary Note 4.1).

**Table 2:** Analysis of data of DelSontro et al.^7^ for the 7 lakes investigated by Günthel et al.^5^.

Lake	CH_4,av_ (µM)	*A*_surf_ (km^2^)	*d*_SML_ (m)	*R*_AV_ (m^−1^)	Prevailing biological process	*R*_CH4_	NOMC (%)
Beauchene	0.036	17	5	0.0099	Production	1.24	19
Champlain	0.089	1269	10	0.0011	Oxidation	0.92	−9
Camichagama	0.025	26	7	0.0081	Production	1.21	17
Nominingue	0.067	22	5	0.0087	Production	1.22	18
Ontario	0.032	19,009	12	0.0003	Production	1.22	18
Simard	0.040	170	10	0.0031	Production	1.41	29
St.-Jean	0.009	1065	5	0.0012	—	—	—

The relative decrease/increase due to oxidation/production in the SML is given by *R*_CH4_ − 1. The contribution of net oxic production to diffusive CH_4_ emissions is NOMC = (*R*_CH4_ − 1)/*R*_CH4_. All data except for *R*_AV_ and NOMC are from DelSontro et al.^7^. Results on all lakes and additional information are provided in Supplementary Table 5 (Supplementary Note 4.1).

*CH*_*4,av*_ average CH_4_ concentration in the SML, *A*_*surf*_ surface area, *d*_*SML*_ depth of the surface mixed layer SML, *R*_*AV*_ *A*_sed_/*V*_SLM_ assuming a sediment slope of 5°, *A*_*sed*_ sediment area in the SML, *V*_*SLM*_ volume of the SML, *R*_*CH4*_ ratio of total emissions to total littoral flux (Supplementary Note 4).

DelSontro et al.^7^ compared observations of the spatial distribution of CH_4_ and δ^13^C of CH_4_ in the SML of lakes with results from numerical simulations and provided estimates on the contribution of net oxic methane production in relation to a reference condition without biological processes. Their numerical model^7^ assumes steady state and includes as sources for emissions only the CH_4_ flux from the littoral and net oxic CH_4_ production. The total emission for the reference condition therefore corresponds to the total flux from the littoral, *F*_litt,tot_. Del Sontro et al.^7^ analyzed the impact of biological processes as fractional increase or decrease, *f*_biol_, of the CH_4_ concentrations relative to the reference condition without oxidation. Figure 4 and Supplementary Table 8 of ref. ^7^ provide values on *f*_biol_ + 1, denoted here as *R*_CH4_ (see Supplementary Note 4.1). Assuming that CH_4,equ_ is negligible compared to the CH_4_ concentrations in the SML, *R*_CH4_ can be interpreted as the ratio of the total CH_4_ emission *F*_surf,tot_ to the emission under reference conditions *F*_litt,tot_, thus *R*_CH4_ ≈ *F*_surf,tot_/*F*_litt,tot_ = (NOM + *F*_litt,tot_)/*F*_litt,tot_ = NOM/*F*_litt,tot_ + 1. The ratio of NOM to *F*_surf,tot_ is given by NOMC = (*R*_CH4_ − 1)/*R*_CH4_ (for details and further analyses, see Supplementary Note 4.1).

DelSontro et al.^7^ provided *R*_CH4_ for six of the seven lakes investigated by Günthel et al.^5^. In these 6 lakes, NOMC is <20% and is negative in Lake Champlain indicating dominance of oxidation in this lake, which is consistent with ref. ^7^ (Table 2).

## Interpretation of the results of our re-analyses

The results on NOMC suggest that net production of CH_4_ is not the dominant source of the CH_4_ emissions from the lakes investigated but fluxes of CH_4_ from shallow water regions (Fig. 1). NOMC is <50% in all lakes except in the South Basin of Lake Stechlin in 2017 where NOMC is unrealistically high because the average *F*_sed_ in the SML is most likely substantially underestimated (the average *F*_sed_ used by, e.g., Günthel et al.^5^ for this basin in 2014, 2016, and 2018 was 5–18 times larger than *F*_sed_ used for 2017, see Supplementary Note 3.3). On average NOMC is 10% (mean of all lakes using for Lake Stechlin the upper limits of 2016, i.e., 37 and 33%, for South and North Basin, respectively) and is 16% for the 6 lakes with lowest *A*_sed_/*V*_SML_. NOMC does not significantly increase with *A*_surf_ or *A*_sed_/*V*_SML_ (correlation using the same data as for the mean of all lakes: *R*^2^ = 0.005, *p* = 0.8, and *R*^2^ = 0.07, *p* = 0.3, respectively). Hence, there is no support for the hypothesis that net oxic CH_4_ production contributes a major fraction of the CH_4_ emitted from the lakes investigated or increases in importance with increasing lake size or decreasing *A*_sed_*/V*_SML_. The latter even suggests that NOMC is negligible.

However, the results of our analysis cannot be taken as proof that NOM is negligible or, in contrast, as a confirmation that NOM contributes up to 37% to CH_4_ emissions, because the uncertainty of the estimated NOMC is high: assessing the relevance of oxic methanogenesis from mass balance approaches involves the difference of comparative large contributions, i.e., surface emissions and sediments fluxes, which both have a large uncertainty. In particular, basin-wide average sediment fluxes from the littoral are not well constraint by estimates based on a single sediment core as in Lake Hallwil and Lake Stechlin, which becomes obvious from the comparison of sediment fluxes in the South Basin of Lake Stechlin derived from mesocosm experiments and the CH_4_ pore water technique applied to a single core (Fig. 1, data from ref. ^11^, and Supplementary Note 3.3). Furthermore, closing mass balances of CH_4_ requires that the components of the mass balance are measured at the same temperature, because CH_4_ fluxes are temperature dependent^15^, and at the same time and not several months or even years apart as in Donis et al.^6^ and Günthel et al.^5^, respectively.

The investigations of Encinas Fernadez et al.^8^, DelSontro et al.^7^, and Peeters et al.^9^ are based on the spatial distribution of CH_4_ and consistently show that the observed horizontal distribution patterns of CH_4_ in the SML require a large source of CH_4_ in the shallow water region to explain the typically enriched concentrations in near shore zones. Spatially averaged CH_4_ concentrations in the SML are not correlated with *A*_surf_ but with *A*_sed_/*A*_surf_^8^ implying that total emissions are proportional to *A*_sed_ in the SML and that the littoral zone must therefore be an important source of CH_4_ emissions^8^. The seasonal change in the horizontal distribution pattern of CH_4_ in the SML and of the overall emission of CH_4_ can be explained by a temperature-dependent sediment flux^9^. However, the quantification of sediment fluxes and of net CH_4_ production using inverse modeling of spatial distributions of CH_4_^7,9^ requires estimates of horizontal turbulent diffusion coefficients, which are unfortunately highly uncertain. Combining inverse modeling of spatial distributions, isotope measurements, and full mass balance approaches is not only a promising avenue to further constrain the relevance of oxic methanogenesis in lakes but also requires information on sediment fluxes, which appear to be the most uncertain component in the studies so far.

Global emissions from lakes are dominated by emissions from small- and medium-sized lakes. Lakes with *A*_surf_ < 1 km^2^ contribute ~84% and lakes with *A*_surf_ > 1 km^2^ only ~16% of the global diffusive CH_4_ emissions from lakes (Supplementary Table 1.2. in ref. ^16^). The contribution of oxic methanogenesis to global diffusive CH_4_ emissions therefore depends on NOMC in small- and medium-sized lakes rather than on NOMC in large lakes. There seems to be consensus that diffusive emissions from lakes <1 km^2^ are dominated by fluxes from littoral zones. Hence, improving the understanding and quantification of the sources of CH_4_ in littoral zones appears to be particularly important for predicting the impact of changing conditions in lakes on the global CH_4_ budget.

## Supplementary information

Supplementary Information

## Data Availability

All relevant data are available from the tables in the supplement and the data sources cited but can also be requested from the authors.
