# Supplementary material for: Oxic methanogenesis is only a minor source of lake-wide diffusive CH4 emissions from lakes
Source: Nat Commun. 2021 Feb 22;12:1206. doi: 10.1038/s41467-021-21215-2 (PMC7900097; doi:10.1038/s41467-021-21215-2)
Supplement: Supplementary file 1 — Supplementary Information [file 41467_2021_21215_MOESM1_ESM.pdf]

Supplementary Information for

Oxic methanogenesis is only a minor source of lake-wide diffusive CH_4 emissions from lakes.

F. Peeters and H. Hofmann

The supplementary information consists of Supplementary Notes 1-4:

1. ***NOM* and *NOMC*: Properties for the investigation of the contribution of biological processes to overall CH_4 emissions from lakes**
2. **Detailed evaluation of the data from Lake Hallwil**
   2.1 CH_4-fluxes from the sediments in the SML of Lake Hallwil
   2.2 Surface emissions in Lake Hallwil
   2.3 NOM and NOMC in Lake Hallwil
3. **Re-analysis of the data from Lake Stechlin**
   3.1 Estimation of sediment fluxes from mesoscosm experiments
   3.2 NOMC in Lake Stechlin
   3.3 Comment on the sediment flux (South basin 2017) estimated by Hartmann et al. 2020[1]
4. **Evaluation of the data from DelSontro et al. 2018[2]**
   4.1. NOMC for the lakes studied by DelSontro et al. 2018 [2]
   4.2. The ratio A_{sed}/V_{SML} for the lakes investigated by DelSontro et al. 2018[2]

1. *NOM* and *NOMC*: Properties for the investigation of the contribution of biological processes to overall CH_4 emissions from lakes

Net-production of methane in oxic waters, *NOM* (**N**et **O**xic **M**ethane production), is estimated from the difference between the total diffusive CH_4 emissions from the lake surface, $F_{surf,tot}$, and the total flux from the sediments in the SML, $F_{sed,tot}$, i.e. $NOM = F_{surf,tot} - F_{sed,tot}$. This procedure neglects processes contributing to the mass balance but allows for a consistent comparison of the observations from Lake Hallwil and Lake Stechlin with the results on "other lakes"[3] derived from data of DelSontro et al. 2018[2]. The processes neglected here but considered by Günthel et al. 2019[3], are vertical transport of CH_4 to the SML and the dissolution of microbubbles. Vertical transport of CH_4 is a small source of CH_4 in the SML of Lake Hallwil and Lake Stechlin (Günthel et al. 2019[3]) and was neglected in the study of DelSontro et al.2018[2]. Because DelSontro et al. 2018[2] do not provide data on the vertical distribution of CH_4 or on vertical diffusivities it is impossible to include the vertical flux of CH_4 in the CH_4 mass balance. The dissolution of CH_4 microbubbles was not measured in any of the studies investigated and is a highly uncertain source to the CH_4 mass balance. Microbubbles were considered by Günthel et al. 2019[3] only in case of Lake Hallwil, but not in Lake Stechlin or in the lakes of DelSonto et al. 2018[2]. Because both processes neglected by us are sources of CH_4 in the SML, the values for *NOM* presented by us are conservative upper limits for net production of CH_4 in the SML. The contribution of *NOM* to overall diffusive CH_4-emission is defined as $NOMC = NOM / F_{surf,tot}$.

Note, that our analysis is based on net production of CH_4, *NOM*, and not on gross production of CH_4 as in Günthel et al. 2019[3]. Because independent measurements of methane oxidation are not available in the datasets analyzed, a data-based closure of the mass balance provides only net production, whereas gross production depends on the methane oxidation assumed. E.g. at zero net-production gross production and oxidation of CH_4 are equal and the value of gross production depends entirely on the choice of the value for CH_4 oxidation. It is therefore advantageous to use *NOM* instead of gross production for a data based assessment of the relevance of biological processes for overall CH_4 emissions from lakes (see also DelSontro et al. 2018[2]).

2. Detailed evaluation of the data from Lake Hallwil

2.1. CH_4-fluxes from the sediments in the SML of Lake Hallwil

Donis et al. 2017[4] and Günthel et al. 2019[3] determined the CH_4-flux from sediments into the water column, F_{sed}, in the SML of Lake Hallwil by averaging F_{sed} estimates from two sediment cores. One of these cores was collected at 3 m and the other at 7 m water depth. Because the SML extends from 0 to 5 m water depth only the core from 3 m water depth is from the SML. $\delta^{13}C$ of the CH_4 in the pore water of the cores from 3 m and 7 m differs substantially between the two cores (see Figure 5b in [4]) suggesting that production and oxidation of CH_4 in the core from the SML, i.e. from 3 m, and the core from 7 m are not the same, probably at least partly due to higher temperatures in the SML. Clearly, estimates of sediment fluxes in the SML should be based on cores suspect to the conditions within the SML, i.e. on the core collected at 3 m water depth.

Donis et al. 2017[4] estimated F_{sed} from the CH_4 in the pore water of sediment cores by assuming steady state conditions and Fick's first law of diffusion: $F = - D\ dC/dz$, whereby D is the molecular diffusion coefficient and dC/dz the concentration gradient as function of depth. Donis et al. 2017[4] applied this concept by utilizing the vertical profile of pore water concentrations of CH_4 and by taking into account the modification of the molecular diffusion coefficient by tortuosity and porosity in sediments. As Fick's first law is a local law providing the flux at the location at which the gradient is measured, the calculation of the flux of CH_4 from the sediment into the water column requires an estimation of the CH_4-gradient in the pore water at the sediment surface. Peeters et al. 2019[5] have shown that the concentration gradient near the sediment surface, i.e. within the top 3 cm of the sediment core, is 1.7 times larger than the gradient used by Donis et al. 2017[4] (Supplementary Figure 1, blue line versus red line). Consequently, the CH_4-flux out of the sediment consistent with the physical process assumed is 1.7 times larger than the F_{sed} of 1.6 mmol m^{-2} d^{-1} published by Donis et al. 2017[4], i.e. F_{sed} = 2.8 mmol m^{-2} d^{-1} for the sediment core collected at 3 m water depth (Peeters et al. 2019[5]).

An additional source of uncertainty in the estimate the F_{sed} is the diffusivity in the pore water of the sediment which assumes homogeneous conditions within the upper few centimeters of the sediment.

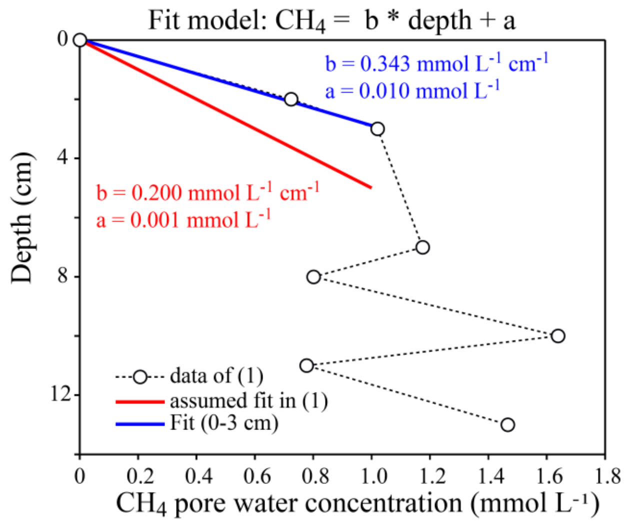

Supplementary Figure 1: CH_4 pore water concentrations measured in a sediment core in Lake Hallwil collected on 29th of September 2016 at 3 m water depth.
Data are digitized from Figure 5 in Donis et al. 2017[4]. The vertical gradient of the pore water CH_4 concentration obtained by a linear fit to the data from the top 3 cm of the sediment core is $3.4 \cdot 10^4$ mmol m^{-4} (blue line), which is 1.7 times larger than the gradient $2.0 \cdot 10^4$ mmol m^{-4} used by Donis et al. 2017[4] and Günthel et al. 2019[3] (red line). The figure is identical to Supplementary Figure 8 in Peeters et a. 2019[5].

2.2. Surface emissions in Lake Hallwil

Wind based models and chamber measurements: Günthel et al. 2019 and Donis et al. 2017 have mistakenly used gas transfer coefficients for CH_4-fluxes

We have calculated gas transfer coefficients of CO_2 at 20°C, k_{600}, and CH_4-fluxes from the lake surface, F_{surf}, in Lake Hallwil using different wind based models for k_{600} (Table 1). CH_4-fluxes were obtained from $F_{surf}(T) = k_{CH4}(T) \cdot (CH_4 - CH_{4,equ}(T))$, and k_{CH4} is the gas transfer coefficient for CH_4 determined from $k_{CH4}(\mathrm{T}) = k_{600} \cdot (S_{CH4}(T)/600)^{\alpha}$ with $\alpha = -2/3$ if $U_{10} \leq 3.7$ m s^{-1} and $\alpha = -1/2$ if $U_{10} > 3.7$ m s^{-1}. T is the water temperature, S_{CH4} the Schmidt number of CH_4 (S_{CH4} (20°C) ~616), and U_{10} the wind speed. $CH_{4,equ}(T)$ is the CH_4 concentration in equilibrium with the atmosphere (0.003 mmol m^{-3} at 20°C in Lake Hallwil[5]). Because $CH_{4,equ}$ is very small compared to the average surface CH_4 concentration ($CH_{4,av}$ = 0.3 mmol m^{-3} , Donis et al. 2017 [4]) used in the flux calculations[3,4], it has no significant effect on the results of F_{surf}.

According to Günthel et al. (pers. communication of supplementary material) they considered water temperature only for $CH_{4,equ}$ but not for k_{CH4}. Hence, the surface fluxes are essentially fluxes evaluated at 20°C, F_{surf} (20°C). At 20°C the values of k_{600} and k_{CH4}(20°C) are almost the same (Table 1). Note that in their mass balance for Lake Hallwil Donis et al.

2017[4] and Günthel et al. 2019[3] compare surface emissions from April to August with a sediment flux determined from a sediment core collected on the 29th of September 2016 when water temperature was ~20°C[5]. As CH_4-fluxes are temperature dependent[6], the mass balance should be based on fluxes measured not only at the same time but also at the same temperature. The inconsistent design of the experiment by Donis et al. 2017[4] may be, at least partly, compensated by using 20°C in the calculation of the surface fluxes[5].

The results of our calculations demonstrate that the values for the CH_4-fluxes at the lake surface provided by Donis et al. 2017[4] and by Günthel et al. 2019[3] are in fact values for gas transfer coefficients but not CH_4-fluxes F_{surf} (Table 1). This conclusion is supported by the perfect agreement of the values published by Günthel et al. 2019[3] for F_{surf} with the values of k_{600} and k_{CH4} determined by us (Table 1). Our calculations can easily be confirmed using the average wind speed, because the applied models of k_{600} depend linearly on wind speed. The wind data provided to us by Günthel et al. 2019[3] cover the time period from the 15th of April to the 28th of July 2016 and the average wind speed was 1.69 m s^{-1}. Thus, the Hallwil relationship, i.e. the model derived by Donis et al. 2017[4] from their floating chamber measurements specifically for Lake Hallwil, gives: $k_{600} = 2 \cdot U10 = 3.4$ cm $hr^{-1} = 0.8$ m d^{-1} and the model of MacIntyre et al. 2010[7] for positive buoyancy flux gives $k_{600} = 1.74 \cdot U10 - 0.15 = 2.8$ cm $hr^{-1} = 0.7$ m d^{-1} (Table 1). Note that k_{600} and k_{CH4} are essentially the same at 20°C. The values published by Donis et al. 2017[4] as CH_4-fluxes are very similar to these k_{CH4} and therefore also do not represent CH_4-fluxes but gas transfer coefficients.

Apparently, Günthel et al. 2019[3] and Donis et al. 2017[4] have erroneously missed to multiply the gas transfer coefficients with the difference between surface concentration and equilibrium concentration of CH_4. Considering that k_{600} and k_{CH4}(20°C) are very similar (Table 1) and that the atmospheric equilibrium concentration is very small (i.e. $CH_{4equ} = 0.003$ mmol m[3],[5]) the factor missing in the calculations of Donis et al. 2017[4] and Günthel et al. 2019[3] is essentially the CH_4 concentration at the lake surface, i.e. 0.3 mmol m^{-3} (Donis et al. 2017[4]). Consistently, the correctly calculated CH_4-fluxes are ~3.3 times smaller than the values published by Donis et al. 2017[4] and Günthel et al. 2019[3] (Table 1, see also Peeters et al. 2019[5]).

The analysis above clarifies that Donis et al. 2017[4] and Günthel et al. 2019[3] apparently have mistaken gas transfer coefficients for CH_4-fluxes in their calculations using empirical wind based models. Donis et al. 2017[4] explicitly stated that the values obtained from their floating chamber measurements agree well with their estimates from the wind based model of MacIntyre et al. 2010[7] for positive buoyancy flux. "*Average flux (April–August 2016) is equal*

to 0.8 ± 0.2 mmol m^{-2} d^{-1} *from MacIntyre relationship for positive buoyancy and to 0.6 ± 0.3 mmol* m^{-2} d^{-1} *from chamber measurements. The latter, not significantly different from the wind-based relationship, was used for the mass balance"* [4]. Günthel et al. 2019[3], co-authored by D. Donis, claim for the "MacIntyre relationship for positive buoyancy[7]" an average value of 0.7 for F_{surf}, but in fact, 0.7 is the average value for the gas transfer coefficient k_{CH4} in unit m d^{-1} (0.7 m d^{-1} see Table 1) and F_{surf} for this model is 3.3 times smaller (0.21 mmol m^{-2} d^{-1} see Table 1). The value published by Donis et al. 2017[4] is slightly larger than 0.7, probably because they averaged CH_4-fluxes divided by $CH_{4,av}$, which does not result in exactly the same value as the average k_{CH4}. Independent of this speculation on the procedure applied by Donis et al. 2017[4], their value is incompatible with F_{surf} but closely agrees with the gas transfer coefficient, as is obvious in the case of Günthel et al. 2019[3]. The good agreement between the values from the MacIntyre model for positive buoyancy flux[7] and the values from the chamber measurements suggests that the values from the chamber measurements are not gas fluxes but gas transfer coefficients.

Donis et al. 2017[4] illustrate in their Supplementary Figure 4[4] that their floating chamber measurements agree well with the "Hallwil relationship", which they derived from their chamber measurements specifically for the conditions in Lake Hallwil (Supplementary Figure 2A). Supplementary Figure 2 clarifies that the estimates from wind based models depicted in Supplementary Figure 2A are not CH_4-fluxes as was suggested by the labeling of Donis et al. 2017[4], but are in fact gas transfer coefficients (Supplementary Figure 2A,B). The comparison of Supplementary Figure 2A-C indicates, that Donis et al. 2017[4] have generally compared gas transfer coefficients and not CH_4-fluxes and supports the argument that their values from the chamber measurements refer to gas transfer coefficients and not to CH_4-fluxes. In this case, the true CH_4-fluxes from the chamber measurements are 3.3 times smaller than those used by Donis et al. 2017[4] and Günthel et al. 2019[3] and only then agree well with the CH_4-fluxes obtained from the model of MacIntyre et al. 2010[7] for positive buoyancy flux.

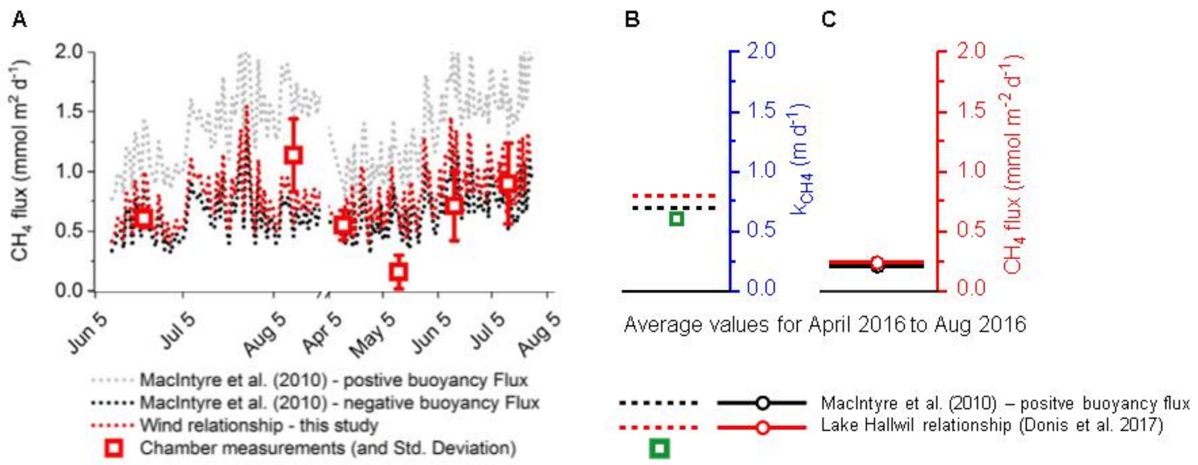

Supplementary Figure 2: Comparison of wind model estimates and floating chamber measurements from Lake Hallwil by Donis et al. 2017[4]. (A) Redrawn from Supplementary Figure 4 of Donis et al. 2017[4]. (B) and (C) Average values of k_{CH4} and of the CH_4 flux, respectively, correctly calculated for the respective wind based models (see Table 1 of the main text). The average value of the chamber measurements of Donis et al. 2017[4] is also depicted in (B) as the most plausible interpretation of the data. The averages are for the time period from April 2016 to August 2016. Note that in Supplementary Figure 4 of Donis et al. 2017[4], i.e. panel A, the first section on the x-axis is for 2015 and the second for 2016 and in the legend the MacIntyre 2010 models[7] should be exchanged (k_{CH4} and gas fluxes are larger for negative than for positive buoyancy fluxes).
The original caption to panel (A) reads: "*Lake Hallwil surface CH_4 fluxes measured with floating chambers (red squares ± SD) and estimated based on different k_{600}, i.e. wind speed dependent water/air gas transfer coefficient between June 2015 and August 2016 (black dashed line) and coefficient from MacIntyre et al. 2010 for positive and negative buoyancy flux. The two chamber measurement series from 12 August 2015 and 15 May 2016 stand out of the wind-based estimates as they were obtained during particularly windy, cool day and a very warm day with little wind, respectively.*" (Donis et al. 2017)[4].

The Hallwil relationship was derived by Donis et al. 2017[4] from their chamber measurements in Lake Hallwil. The establishment of this Hallwil relationship required that Donis et al. 2017[4] calculated gas transfer coefficients from their chamber measurements. Supplementary Figure 2B depicts the average of the values from the chamber measurements and the average of the gas transfer coefficient k_{CH4} obtained from the wind model Hallwil relationship for April to August 2016. During this time period, which was used in the mass balance for Lake Hallwil[4], the average value from the chamber measurements was affected by the specifically low value in May[4] and therefore was slightly smaller than the average value of the k_{CH4} from the Hallwil relationship. Considering the entire time period depicted in Supplementary Figure 2A the average of the values from the chamber measurements and of k_{CH4} calculated from the Hallwil relationship agree very well. It is important to note that the values depicted for the Hallwil relationship Supplementary Figure 2B are gas transfer coefficients and chamber measurements therefore only agree with the Hallwil relationship derived from the chamber measurements, if the chamber measurements depicted in

Supplementary Figure 2A also represent gas transfer coefficients and not F_{surf}. If the values from the chamber measurements are however taken as CH_4-fluxes as in Donis et al. 2017[4], Günthel et al. 2019[3] two questions require an answer: (i) Why does the model derived from the chamber measurements, i.e. the Hallwil relationship, provide substantially smaller CH_4-fluxes than the chamber measurements themselves, i.e. about 0.3·times the chamber values; (ii) Why does this factor 0.3 agree so very well with the value of the surface concentration of CH_4 that was already missing in the flux calculations of the wind based models?

The Hallwil relationship as most reliable estimate of the average k_{600} and F_{surf}

The mass balance in Lake Hallwil was based on average CH_4 surface fluxes for the time period between April and August. For this time period the average value of the transfer coefficient obtained from the Hallwil relationship is larger than the average value obtained from the chamber measurements (Supplementary Figure 2B) because 1 of the 4 available chamber measurements from 2016 is exceptionally low: Donis et al. 2017[4] state: "*15 May 2016 stand out of the wind-based estimates as they were obtained during … a very warm day with little wind"* [4]. Hence, the chamber measurements underestimate the average k_{CH4} for April to August 2016. The Hallwil relationship, being based on the chamber measurements and providing a better temporal resolution than the chamber measurements, is therefore considered here as the most reliable means for the estimation of the average k_{CH4} for April to August 2016, i.e. k_{CH4} = 0.8 m d^{-1} (Table 1).

The reliability of the Hallwil relationship was confirmed by Günthel et al. 2019[3] and by Hartmann et al. 2020[1] comparing different estimates of surface fluxes in the South Basin of Lake Stechlin.

2.3 NOM and NOMC in Lake Hallwil

The mass balance of Donis et al. 2017[4] and Günthel et al. 2019 [3] for Lake Hallwil considers CH_4 emissions at the lake surface, CH_4 fluxes from sediments, and additional contributions that were small (vertical turbulent transport of CH_4) or were not measured and are highly uncertain (dissolution of microbubbles). These additional contributions are neglected in the following (explanation see supplement A). Because the neglected terms are sources of CH_4 in the SML, the values estimated by us are upper limits of the NOM. Note that methane oxidation, which was not measured independently, is not required because we consider net production and not gross production. The results of the mass balance are

summarized in Supplementary Table 1. The balance is based on the Hallwil relationship (explanation see above) and indicates that net-production of CH_4 contributes ~17% to overall emissions, i.e. NOMC = 17%.

Supplementary Table 1: Estimation of NOMC in Lake Hallwil.
The columns provide CH_4 fluxes at the lake surface, F_{surf}, and total CH_4 emissions, $F_{surf,tot}$, for different methods for estimating surface fluxes, the total CH_4 sediment flux, $F_{sed,tot}$, estimated from the sediment flux from the core collected at 3 m water depth, F_{sed} = 2.8 mmol m^{-2} d^{-1}, net-production of methane in oxic waters $NOM = F_{surf,tot} - F_{sed,tot}$, and the contribution of NOM to the total methane emission $NOMC = NOM / F_{surf,tot}$. The surface area of Lake Hallwil is $A_{surf} = 9.9 \cdot 10^6$ m^2 and the area of the sediments in the SML (0-5 m water depth) is $A_{sed} = 0.7 \cdot 10^6$ m^2 (Peeters et al. 2019[5], confirmed by Günthel et al. 2019[3]).

	F_{surf} (mmol m^{-2} d^{-1})	$F_{surf,tot}$ (mol d^{-1})	$F_{sed,tot}$ (mol d^{-1})	NOM (mol d^{-1})	NOMC (%)
**Method for estimation emissions**					
**Wind models:**					
Lake Hallwil relationship	0.24	2376	1960	416	17
MacIntyre et al. 2010[7] positive buoyancy flux	0.21	2079	1960	119	5
**Chamber measurements:**					
Values assumed to be transfer coefficients	0.18	1782	1960	-178	-10
Interpreted as gas fluxes (Donis et al. 2017)[4]	0.6	5940	1960	3980	67

3. Re-analysis of the data from Lake Stechlin

3.1. Estimation of sediment fluxes from mesocosm experiments

The sediment flux in the mass balances for Lake Stechlin by Günthel et al. 2019[3] are underestimated and oxic metanogenesis is therefore overestimated. Sediment fluxes were calculated by assessing oxic production from mesocosm experiments and by using these estimates of CH_4-production in a mass balance for the lake closing the balance to determine the sediment flux. CH_4 surface fluxes from the mesocosms were utilized to calculate CH_4 production within the mesocosms. However, F_{surf} from the mesocosm was overestimated because the gas transfer coefficient used in the mesocosms was taken to be the same as determined for the open water of the lake, but the turbulence in the mesocosm is substantially lower than in the open water.

The effect of turbulence on the gas transfer coefficient can be assessed from the scaling relation $k \sim Sc^{-1/2} (\nu \cdot \varepsilon)^{¼}$, where Sc is the Schmidt number, ν the kinematic viscosity and ε the energy dissipation. The scaling relation was derived from the eddy cell model of gas transfer at the air–sea interface[8] and can also be obtained from dimensional arguments scaling the thickness of the diffusive boundary in a diffusive boundary layer approach using the Bachelor length scale[9]. MacIntyre et al. 2010[7] have shown that the scaling relation $k \sim \varepsilon^{¼}$ agrees well with transfer coefficients measured with the eddy correlation technique. The calculation of k_{600} directly from the scaling relation requires that ε is measured very near the lake surface. However, the scaling relation indicates that the relative size of k_{600} is proportional to the relative intensity of turbulence characterized by ε.

Values on energy dissipation ε are available for open lake water and a mesocosm in Lake Stechlin at a vertical resolution of 0.05 m from 1.05 m water depth on downwards (data source to Supplementary Figure 8 in Günthel et al. 2019[3]). We considered the uppermost 1 m of measurements, i.e. 20 values from 1.05 to 2.00 m. The ratio between the average ε in the lake and the average ε in the mesocosm is ~ 5 at high wind and ~ 6 at low wind conditions. However, the uppermost values (1.05 m water depth) seem to be outliers in lake and mesocosm: At 1.05 m water depth ε in the lake is 70 times larger at low than at high wind conditions whereas below this first values ε is typically smaller at low than at high winds. . At high winds the value of ε in the mesocosm is 56 time larger at 1.05 m water depth than at 1.10 m. The average ε calculated without the values from 1.05 m, depth i.e. using the 19 values from 1.10 m to 2.00 m depth, suggests that energy dissipation in the lake is ~10 times larger than in the mesocosm (~10 at high wind and ~9 at low wind conditions).

The data clearly indicate that turbulence in the open water of the lake is substantially larger than in the mesocosm and therefore strongly suggest that the gas transfer coefficient should be smaller in the mesocosm than in the lake. In the following, we estimate k_{600} in the mesocosm by scaling k_{600} for the open water using the relation $k_{600} \sim \varepsilon^{1/4}$ and the ratio between ε in the lake and ε in the mesocosm, i.e. a ratio of 5 as lower and of 10 as upper limit. Hence, k_{600} for the lake must be scaled by $5^{-1/4} = 0.67$ and $10^{-1/4} = 0.56$ to provide upper and lower bounds, respectively, of k_{600} for the mesocosms. Note that surface fluxes scale by the same scaling factors.

With this scaling approach, which accounts for the difference in turbulence between the mesoscosms and open water of the lake, estimates of net-production become smaller and estimates of sediment fluxes larger than the values provided by Günthel et al. 2019[3]. The corrected sediment flux ranges between 1.8 mmol m^{-2} d^{-1} and 2.0 mmol m^{-2} d^{-1} (Supplementary Table 2).

Supplementary Table 2: Assessment of sediment fluxes from the mesocosm- and lake measurements conducted 2014 in the South Basin of Lake Stechlin

Gas transfer coefficients for the open water from the lake were $k_{600,L}$ = 5.115 cm hr^{-1} (Supplementary Table 4 in Günthel et al. 2019[3]). k_{600} for the mesocosms was calculated from $k_{600,L}$ using the scaling relation $k_{600} \sim \varepsilon^{1/4}$ and the ratio of ε between lake and mesocosm. The fluxes determined from the surface concentrations of the two mesocosms were averaged as in Günthel et al. 2019[3]. Note that the average surface flux (F_{surf} = 0.432 mmol m^{-2} d^{-1}, Supplementary Table 4 of [3]) scales by the same factor as the k_{600}. F_{sed} in the SML was obtained by closing the mass balance for the South Basin and by multiplying the contribution from the sediment with the volume of the SML and dividing by A_{sed} in the SML. The first column lists the values of Günthel et al. 2019[3] that assume the same k_{600} in lake and mesocosms. Morphometry was taken from [3]. Mesosocms: A_{surf}: 63.6 m^2 V_{SML}: 381.6 m^3, Lake: A_{surf} : 1,122,775 m^2. V_{SML}: = 5,726,175 m^3 A_{sed}= 314,775 m^2.

Contributions	**Change of CH_4 per volume ($k_{600,L}$) (nmol m^{-3} d^{-1})**	**Change of CH_4 per volume $k_{600,L}$ *0.67 (nmol m^{-3} d^{-1})**	**Change of CH_4 per volume $k_{600,L}$ *0.56 nmol m^{-3} d^{-1}**
**Mesocosms**			
**Surface flux**	72	48	40
**Diffusion from the thermocline**	1.1	1.1	1.1
**Net production**	70.9	46.9	38.9
**South Basin**			
**Surface flux**	150.7	150.7	150.7
**Diffusion from the thermocline**	3.3	3.3	3.3
**Net production**	70.9	46.9	38.9
**Flux from the sediments**	76.5	100.5	108.5
**Derived properties**			
F_{sed}	1.4 mmol m^{-2} d^{-1}	1.8 mmol m^{-2} d^{-1}	2.0 mmol m^{-2} d^{-1}
NOMC	47%	31%	26%

3.2. NOMC in Lake Stechlin

We used the sediment fluxes from Supplementary Table 2 to re-evaluate the mass balances from Lake Stechlin (Supplementary Table 3), thereby relying on the assumption of Günthel et al. 2019[3] that the sediment flux is the same between the years 2014 and 2018 and in the North and the South basin of Lake Stechlin.

The total CH_4 emissions $F_{surf,tot} = F_{surf} \cdot A_{surf}$ from the surface of the basins were calculated from the surface fluxes for South Basin and North Basin of Lake Stechlin provided by Günthel et al. 2019[3] (Supplementary Table 4 in Günthel et al. 2019[3]). In their mass balance Günthel et al. 2019[3] used averaged fluxes for each year and considered data only from the stratified time period and therefore included only data from June to August. Günthel et al. 2019[3] state: „*The surface methane emission (F_S) was either measured using a flux chamber (all Northeast basin values except on 20th June) or estimated from a wind-based model (all other values) that was developed from the flux chamber measurements and concurrent wind conditions*"[3]. Consistently we use average fluxes from the wind relationship[3] ("Stechlin relationship") for the South Basin (August 2014, June to July 2016) and average fluxes from the chamber measurements for the North Basin (June to July 2016) of Günthel et al. 2019[3] (all data from Supplementary Table 4 of Günthel et al. 2019[3]).

NOMC determined from our estimates of sediment fluxes and emissions shows that even for the lower bound of the sediment flux, i.e. 1.8 mmol m^{-2} d^{-1}, net production contributes less than 40% to the diffusive emissions in all basins and at all times (Supplementary Table 3 below). In the South Basin *NOMC* agrees very well between the years 2014 and 2016 and the average *NOMC* is 29% and 36% for the upper and lower bound of sediment fluxes, respectively. In the North Basin *NOMC* ranges between 26% and 33% and is therefore very similar to *NOMC* in the South Basin.

Supplementary Table 3: Lake Stechlin: *NOMC* estimated from the mass balance of CH_4 in 2014, 2016 and 2018 in the South and North Basin.

Total emissions $F_{surf,tot} = F_{surf} \cdot A_{surf}$ were calculated from the average F_{surf} determined for the North Basin from chamber measurements ($2016_{Chamber}$, June-July 2016; chamber data Supplementary Table 4 of [3]), and for the South Basin from the "Stechlin relationship"[3] derived from chamber measurements (2014, 2016; Data for the South Basin: August 2014 and June-July 2016 from Supplementary Table 4 of [3]); A_{surf} = 1.123 km^2 and A_{surf} = 2.007 km^2 for South and North Basin, respectively[3]. In the North Basin, we also consider an average F_{surf} for June-July 2016 that includes in addition to the fluxes from chamber measurements a flux for the 20th June calculated from the "Stechlin relationship" (2016_{Ch_20J}), which is the flux used by Günthel et al. 2019[3]. The total CH_4 sediment flux $F_{sed,tot} = F_{sed} \cdot A_{sed}$ is calculated from F_{sed} provided in the Supplementary Table 2 and the surface area of the sediment[3]: South Basin: 2014: $A_{sed} = 3.15 \cdot 10^5$ m^2; 2016: $A_{sed} = 3.02 \cdot 10^5$ m^2 (average of A_{sed} in 5 m and 6 m water depth); North Basin 2016: $A_{sed} = 2.65 \cdot 10^5$ m^2 (average of A_{sed} in 5 m and 6 m depth). $NOM = F_{surf,tot} - F_{sed,tot}$ and $NOMC = NOM / F_{surf,tot}$. For 2018 no data were available but Figure 3 in Günthel et al. 2019[3] indicates negative fluxes for the South Basin. Note that the *NOMC* for 2014 are here slightly larger than in Supplementary Table 2 because in this Supplementary Table 3 we have not included CH_4 sources due vertical transport.

**Year of sampling**	F_{surf} (mmol m^{-2} d^{-1})	$F_{surf,tot}$ (mol d^{-1})	$F_{sed,tot}$ F_{sed} = 1.4 mmol m^{-2} d^{-1} (mol d^{-1})	**NOM** (mol d^{-1})	**NOMC** (%)	$F_{sed,tot}$ F_{sed} = 1.8 mmol m^{-2} d^{-1} (mol d^{-1})	NOM (mol d^{-1})	**NOMC** (%)	$F_{sed,tot}$ F_{sed} = 2.0 mmol m^{-2} d^{-1} (mol d^{-1})	NOM (mol d^{-1})	**NOMC** (%)
**South Basin**											
2014	0.769	863	441	422	49	567	296	**34**	630	233	**27**
2016	0.772 (± 24%)	867	423	444	51	544	323	**37**	605	262	**30**
**North basin**											
$2016_{Chamber}$	0.356 (± 25%)	715	372	343	48	478	237	**33**	531	184	**26**
2016_{Ch_20J}	*0.472 (± 62%)*	*942*		*570*	*61*		*464*	*49*		*411*	*44*

The mean value of the emission from the North Basin in 2016 used by Günthel et al. 2019[3] is more than 30% larger than the average emission obtained from the chamber measurements. Apparently they have combined the fluxes from the chamber measurements with an additional value for June 20th calculated from their wind model. However, this value from 20th of June (1.05 mmol m^{-2} d^{-1}) exceeds the average flux from all chamber measurements (0.36 ± 0.09 mmol m^{-2} d^{-1}) by a factor of ~3 (Supplementary Figure 3A). Including this single exceptionally high value from June 20th causes that the average emission from June to July 2016 is 30% larger than the average emission calculated from the chamber measurements for the same time period.

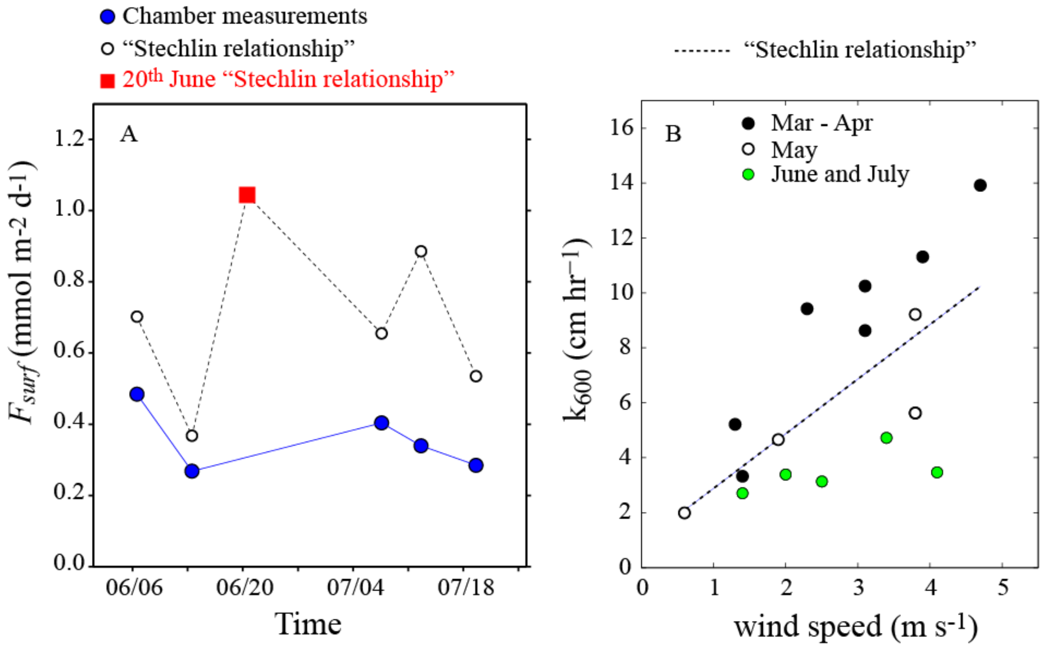

Supplementary Figure 3: CH_4 surface fluxes and k_{600} in the North Basin of Lake Stechlin measured with chambers and estimated with the Stechlin relationship of Günthel et al. 2019[3].
(A) CH_4 surface fluxes derived from the Stechlin relationship substantially overestimate fluxes from chamber measurements. Günthel et al. 2019[3] combined the fluxes from the chamber measurements with the flux from the 20th of June for their estimate of the average surface flux. (B) The Stechlin relationship was obtained by a linear regression considering all k_{600} derived from chamber measurements as function of wind speed. The slope of k_{600} versus wind speed differs between unstratified (March-April) and stratified conditions (June -July).

The strong deviation between the average flux from the chamber measurements and the value for June 20th can be explained by the fact that the wind model, i.e. the "Stechlin relationship" developed by Günthel al. 2019[3] substantially overestimates surface fluxes during the stratified period. Günthel et al. 2019[3] derived the "Stechlin relationship" from a linear regression of k_{600} determined from their chamber measurements as function of wind speed. In the linear regression Günthel et al. 2019[3] considered all available data from March

to July in the North Basin of Lake Stechlin. However, k_{600} not only depends on wind speed but also on convective mixing indicated by the surface buoyancy flux[7]. Consistently, the slope of k_{600} versus wind speed is substantially larger during unstratified periods typically characterized by intense vertical mixing than during stratified conditions (Supplementary Figure 3B). The “Stechlin relationship” therefore substantially overestimates F_{surf} during June and July 2016 (Supplementary Figure 3A), which is the time period used for the mass balances[3].

Because the value for the flux from the 20th of June is clearly overestimated by the Stechlin relationship we rely in the further analysis on the results based only on the chamber measurements. Nevertheless, for completeness we also provide *NOMC* obtained from the estimate of the average emission from the North Basin that includes the high value on the flux from the 20th of June (Supplementary Table 3 indicated as 2016_{Ch_20J}, also included in Figure 1 and Supplementary Figures 4 and 5).

Note that the overestimation of the surface fluxes by the “Stechlin relationship” has also consequences for the estimated sediment fluxes, because these were determined based on surface fluxes from 2014 that were derived from the “Stechlin relationship”. Using a wind model “Stechlin stratified” based on a linear regression of the flux chamber data versus wind speed considering only the stratified period from the 24th of May to the end of July (k_{600} = 0.75 *wind speed (m s^{-1}) +1.7 [cm h^{-1}], Supplementary Figure 3B) results in sediment fluxes of 1.2 to 1.3 mmol m^{-2} d^{-1}. However, because the emissions are lower for this Hallwil stratified relationship, *NOMC* for the South Basin turns out to be the same as in Supplementary Table 3. In the North Basin, however, *NOMC* then ranges between 51% and 55%.

3.3. Comment on the sediment flux in the South basin 2017 estimated by Hartmann et al. 2020[1]

Hartmann et al. 2020[1] collected a sediment core from the SML of Lake Stechlin, i.e. from 5 m water depth, and measured a profile of CH_4 in the pore water. Using the same approach as Donis et al. 2017[4] and Peeters et al. 2019[5], Hartmann et al. 2020[1] estimated the flux of CH_4 at ~10 cm depth within the sediment to be ~0.92 mmol m^{-2} d^{-1}. $\delta^{13}C$ of the pore water CH_4 indicates CH_4 oxidation in the upper 10 cm of the core suggesting that the CH_4 flux near the sediment surface is smaller than the estimated CH_4 flux. The gradient of CH_4 in the uppermost 2 cm and 5 cm of this core is only ~8% and ~28%, respectively, of the CH_4 gradient used by Hartmann et al. 2020[1] (pore water data of Hartmann et al. 2020[1]). Assuming

that the sediment properties, i.e. tortuosity and porosity are the same throughout the core, the small near surface gradients implies a sediment flux of ~0.08 mmol m^{-2} d^{-1} and 0.26 mmol m^{-2} d^{-1}, respectively.

It seems unrealistic that such low sediment fluxes are representative for the average CH_4 flux from littoral sediments in the South Basin of Lake Stechlin. The low sediment fluxes are also incompatible with the sediment flux estimated from the mesocosm experiments in 2014, 1.8 mmol m^{-2} d^{-1} to 2.0 mmol m^{-2} d^{-1} and the assumption of Günthel et al. 2019[3] that the sediment flux remains the same between 2014 and 2018 . These arguments suggest that observations from a single sediment core may not provide a reliable estimate of basin wide average sediment fluxes.

However, taking the low sediment fluxes as representative for the average flux from littoral sediments implies that essentially all methane in the SML originates from oxic methanogenesis, i.e. 92 to 97% (Supplementary Table 4). In this case CH_4 concentrations in the shallow zone would be expected to be smaller than in the open water, because in shallow waters with depths smaller than the SML, less CH_4 is produced than in the open water, but losses are similar if gas transfer coefficients are horizontally homogeneous.

Hartmann et al. 2020[1] apparently conducted another set of flux measurements with chambers in the South Basin of Lake Stechlin. They however did not provide these data but only fluxes from a wind model based on these data. The fluxes calculated with this wind model are available from a table column somewhat misleadingly called “floating chamber measurements” (Supplementary Table 1 of Hartmann et al. 2020[1]). Especially at high winds the fluxes from the wind model of Hartmann et al. 2020[1] are substantially larger than fluxes of other wind based models because according to the model of Hartmann et al. 2020[1] k_{600} ~3.4·wind_speed, whereas most other model have a smaller dependence on wind speed.

The mass balance based on the sediment fluxes derived from the CH_4 pore water of the sediment core and the fluxes determined from the wind model of Hartmann et al. 2020[1] (second column of Supplementary Table 1 in [1]) provide *NOMC* ranging from 92% to 97%, respectively (Supplementary Table 4). Note that using the wind model of Hartmann et al. 2020[1] and the sediment fluxes derived from the mesocosm experiments of Günthel et al.2019[3] provide *NOMC* for the South Basin in 2017 (28% to 35%) that are very similar to *NOMC* for the South Basin from 2014 and 2016 (average *NOMC*: 29% to 36%).

Supplementary Table 4: *NOMC* in the South Basin of Lake Stechlin June 2017: Comparison of NOMC calculated from data of Hartmann et al. 2020 and from data of Günthel et al. 2019.

Surface emissions were determined from data of [1] using the wind based model of Hartmann et al. 2020[1] (Hartmann) and the "Stechlin relationship" of Günthel et al. 2019[3] (Stechlin). Sediment fluxes were estimated from CH_4 in the pore water of a sediment core collected in 2017 at 5 m water depth in the South Basin[1] using the CH_4 gradient within the upper 5 cm (Hartmann_{pore_5}) and the upper 2 cm (Hartmann_{pore_2}) of the sediment core, respectively. Upper and lower values of sediment fluxes from the analysis of the mesocosm experiment in 2014 (evaluation see above) were also considered ($\text{Stechlin,}_{meso\_up}$, $\text{Stechlin,}_{meso\_low}$ respectively). Total emissions $F_{surf,tot} = F_{surf} \cdot A_{surf}$ were calculated from the average F_{surf}. $A_{surf} = 1.123\ km^2$ (Günthel et al. 2019[3]) and $A_{sed} = 3.25 \cdot 10^5\ m^2$ (Hartmann et al. 2017[1]). $NOM = F_{surf,tot} - F_{sed,tot}$ and $NOMC = NOM / F_{surf,tot}$.

**Model**	**total CH4 emissions** (mol d^{-1})	**CH_4 sediment flux** (mmol m^{-2} d^{-1})	**total CH_4 sediment flux** (mol d^{-1})	**NOM** (mol d^{-1})	**NOMC** (%)
Hartmann_{pore_5}	1031	0.26	85	946	**92**
Hartmann_{pore_2}	1031	0.08	26	1005	**97**
$\text{Stechlin,}_{meso\_up}$	902	2.0	650	252	28
$\text{Stechlin,}_{meso\_low}$	902	1.8	585	316	35

4. Evaluation of the data from DelSontro et al. 2018[2]

4.1 NOMC for the lakes studied by DelSontro et al. 2018[2]

Günthel et al. 2019[3] re-analyzed data from 7 lakes originally investigated by DelSontro et al. 2018[2] and claim that in these lakes the oxic methane production contributes between 82% to 100% of the total CH_4-emissions. This result appears incompatible with the conclusion of DelSontro et al. 2018[2]: "*Expressed as a fractional increase or decrease in concentration due to biological processes … net impact of biological processes was not trivial, with an average 20% decrease in lakes dominated by oxidation and an average 25% increase in those dominated by a non-littoral CH_4 input*".

It is unclear how Günthel et al. 2019[3] performed their re-analysis of a selected data set from 7 lakes of DelSontro et al. 2018[2]. Günthel et al. 2019[3] state: „*methane concentrations of transect measurements reach a plateau phase at distances (corresponds to equivalent radius) ≥ 2 km..... To estimate the dimension of both major methane sources, first the plateau concentration was integrated over the lakes' equivalent radius (resembling a measure of the oxic methane source), then elevated methane concentration were integrated over the distance of the gradient*"[3]. In nearly half of the lakes re-evaluated by Günthel et al. 2019[3] no data are available at distances > 2 km from shore (see Supplementary Table 5). Günthel et al. 2019[3] also did not explain what they mean by „The distance of the gradient"[3] in their integration.

We therefore also provide values of *NOMC* which are based on the analysis of DelSontro et al. 2018[2] and consider all investigated lakes (Table 2 lists the subset of lake studied by Günthel et al. 2019[3], and Supplementary Table 5 provides analysis and more detailed information for all lakes of DelSontro et al. 2018[2]).

Supplementary Table 5: Summary of the analyses of the data DelSontro et al. 2018[2] .

All data except for R_{AV} and *NOMC* are from DelSontro et al. 2018[2]. D_{max}: Maximum distance from shore for which data were available; *T*: Water temperature; $CH_{4,av}$: Average CH_4 concentration in the SML; A_{surf}: Surface area, d_{SML}: Depth of the SML, R_{AV}: Ratio of sediment area in the SML to volume of the SML assuming a sediment slope of 5° (see Supplementary Note 4.2), R_{CH4}: Ratio of total emissions to total littoral flux. R_{CH4} -1 is the relative decrease / increase due to oxidation / production in the SML. $NOMC = (R_{CH4}\text{-}1)/R_{CH4}$. $NOMC_{CH4,meas}$: Details in Supplementary Note 4.1.

Lake	D_{max} (m)	T (°C)	$CH_{4,av}$ (mmol m^{-3})	A_{surf} (km^2)	d_{SML} (m)	R_{AV} (m^{-1})	Dominant process	R_{CH4}	*NOMC* (%)	$NOMC_{CH4,meas}$ (%)
Beauchene	1300	22.0	0.036	17	5	0.0099	Production	1.24	19	21
Champlain	6700	20.7	0.089	1269	10	0.0011	Oxidation	0.92	-9	-9
Camichagama	1150	19.9	0.025	26	7	0.0081	Production	1.21	17	20
Nominingue	1300	22.1	0.067	22	5	0.0087	Production	1.22	18	19
Ontario	23000	11.8	0.032	19009	12	0.0003	Production	1.22	18	20
Simard	4000	19.9	0.040	170	10	0.0031	Production	1.41	29	31
St..-Jean	10000	15.3	0.009	1065	5	0.0012	-	-	-	-
Achigan	550	23.0	0.131	5.3	4	0.018	Production	1.28	22	22
Croche	80	20.6	0.280	0.07	3	0.17	Production	1.04	4	4
Cromwell	90	20.0	0.800	0.10	2	0.14	Oxidation	0.63	-59	-59
des Ilets	400	21.1	0.182	1.9	2	0.030	Production	1.09	8	8
MacDonald	500	23.0	0.235	3.5	4	0.022	Production	0.79	-27	-27
Morency	200	22.7	0.508	0.26	3	0.084	Production	1.51	34	34
Purvis	185	23.3	0.653	0.19	3	0.099	Production	0.88	-14	-14

DelSontro et al. 2018[2] compared observations of the spatial distribution of CH_4 and $\delta^{13}C$ of CH_4 in the SML with the results from a model considering a littoral source of methane in the SML, emissions from the lake surface, net oxic methane production within the SML, and lateral transport. Vertical transport of CH_4 or other additional sources or sinks in the SML were neglected. DelSontro et al. 2018[2] calculated from their simulation results the fraction of methane oxidized, f_{ox} (equation 22 in the supplement of DelSontro et al. 2018[2]):

$$f_{ox} = 1 - \frac{CH_{4,(\mathrm{all})}}{CH_{4,(\mathrm{wo\ oxi})}} \quad and \quad 1 - f_{ox} = \frac{CH_{4,(\mathrm{all})}}{CH_{4,(\mathrm{wo\ oxi})}} \qquad (1)$$

and f_{ox} is defined to be positive if oxidation is larger than production, $CH_{4,(\mathrm{wo\ oxi})}$ is the concentration that would establish without CH_4 oxidation and $CH_{4,(\mathrm{all})}$ is the concentration considering all processes including oxidation of CH_4. The values on “the relative CH_4 decrease/increase due to oxidation/production” provided by DelSontro et al. 2018[2] in their Supplementary Table 8 and depicted in their Figure 4 correspond to the second expression in equ. 1 and are denoted here as R_{CH4}, i.e. $R_{CH4} = 1 - f_{ox}$. R_{CH4} can also be expressed in terms of the fractional increase due to net production, f_{NOM}, which is defined to be positive if production is larger than oxidation:

$$R_{CH4} = 1 - f_{ox} = 1 + f_{NOM} = \frac{CH_{4,(\mathrm{all})}}{CH_{4,(\mathrm{wo\ oxi})}}. \qquad (2)$$

The model of DelSontro et al. 2018[2] assumes steady state and includes as sources only the CH_4-flux from the littoral and net oxic CH_4 production, *NOM*. At steady state the sum of all sources and sinks of CH_4 must be compensated by overall emissions, $F_{surf,tot}$. The CH_4 concentrations in the SML therefore adjust to values such that the oversaturation of CH_4 provides the CH_4 fluxes required to compensate all sources. In case of the reference condition with zero net production the only source is the flux from the littoral, $F_{litt,tot}$. Therefore, $CH_{4,(\mathrm{wo\text{-}oxi})}$ is smaller than $CH_{4,(\mathrm{all})}$ in the case of positive net production, and is larger than $CH_{4,(\mathrm{all})}$ in the case of a dominance of CH_4 oxidation. The ratio between the flux from the lake surface considering all sources and sinks, $F_{surf.tot}$, and the flux considering only the littoral source neglecting net oxidation, $F_{surf,wo\ oxi}$, is:

$$\frac{F_{surf,tot}}{F_{surf,wo\ oxi}} = \frac{A_{surf} \cdot k_{CH4} \cdot \left(CH_{4(\mathrm{all})} - CH_{4,equ}\right)}{A_{surf} \cdot k_{CH4} \cdot \left(CH_{4,(\mathrm{wo\ oxi})} - CH_{4,equ}\right)} = \frac{CH_{4(\mathrm{all})} - CH_{4,equ}}{CH_{4,(\mathrm{wo\ oxi})} - CH_{4,equ}} \qquad (3)$$

and A_{surf} is the surface area, k_{CH4} the gas transfer coefficient for CH_4, and $CH_{4,equ}$ the atmospheric equilibrium concentration of CH_4. Equ. (3) assumes, as did also DelSontro et al.

2018[2], that k_{CH4} and $CH_{4,equ}$ are horizontally homogeneous. Neglecting $CH_{4,equ}$, which is typically substantially smaller than CH_4 in the surface water, e.g. 2 orders of magnitude in Lake Hallwil and typically an order of magnitude or more in the lakes studied by DelSontro et al. 2018[2] (Supplementary Figure 2 in [2]), the values provided by DelSontro et al. 2018[2] can be interpreted as the ratio of overall emissions to the total flux from the littoral: $R_{CH4} = F_{surf,tot}/F_{litt,tot}$:

$$R_{CH4} = \frac{CH_{4(\text{all})}}{CH_{4,(\text{wo oxi})}} \approx \frac{F_{surf,tot}}{F_{surf,wo\ oxi}} = \frac{F_{surf,tot}}{F_{litt,tot}} \quad (4)$$

Note that at steady state $F_{surf,wo\ oxi} = F_{litt,tot}$ and $F_{surf,tot} = NOM + F_{litt,tot}$ whereby all terms correspond to the respective overall sources and sinks in the SML, typically expressed in mol d^{-1}.

The ratio of net oxic methane production to overall emissions, *NOMC*, can be estimated from R_{CH4}:

$$R_{CH4} \approx \frac{F_{surf,tot}}{F_{litt,tot}} = \frac{NOM + F_{litt,tot}}{F_{litt,tot}} = \frac{NOM}{F_{litt,tot}} + 1 \quad (5)$$

$$NOMC = \frac{NOM}{F_{surf,tot}} = \frac{NOM}{F_{litt,tot}} \frac{F_{litt,tot}}{F_{surf,tot}} = (R_{CH4} - 1)\frac{1}{R_{CH4}} \quad (6)$$

The estimates of *NOMC* for all lakes for which DelSontro et al. 2018[2] presented R_{CH4} are summarized in Supplementary Table 5 and depicted in Figure 1and Supplementary Figures 4 and 5.

The effect of neglecting $CH_{4,equ}$ on the results of *NOMC* can be estimated using the measured average CH_4 in the SML, $CH_{4,meas}$, provided by DelSontro et al. 2018[2].

$$CH_{4,(\text{wo oxi})} = \frac{CH_{4,(all)}}{R_{CH4}} \quad \text{and} \quad CH_{4,(\text{all})} = CH_{4,meas} \quad (7)$$

$$R_{flux,CH4} = \frac{F_{surf,tot}}{F_{litt,tot}} = \frac{CH_{4(\text{all})} - CH_{4,equ}}{CH_{4,(\text{wo oxi})} - CH_{4,equ}} = \frac{CH_{4,meas} - CH_{4,equ}}{CH_{4,meas}/R_{CH4} - CH_{4,equ}} \quad (8)$$

$$NOMC_{CH4,meas} = \left(R_{flux,CH4} - 1\right)\frac{1}{R_{flux,CH4}} \quad (9)$$

$NOMC_{CH4,meas}$ are only slightly larger than *NOMC* (Supplementary Table 5) indicating that neglecting $CH_{4,equ}$ does not introduce a large error.

The analysis of $NOMC_{CH4,meas}$ requires that CH_{4meas} is the average concentrations in the surface water considering the spatial distribution of CH_4. It is not clear whether CH_{4meas} provided by DelSontro et al. 2018[2] represent such spatially weighted averages or are averages

of the values measured. We therefore use *NOMC* in Figure 1 and Supplementary Figures 4, and 5.

Supplementary Figure 4 shows that *NOMC* does not increase with lake size, whereas Figure 1 and Supplementary Figure 5 show, that *NOMC* also does not increase with the ratio A_{sed}/V_{SML} . The ratio A_{sed}/V_{SML} is estimated in the following section.

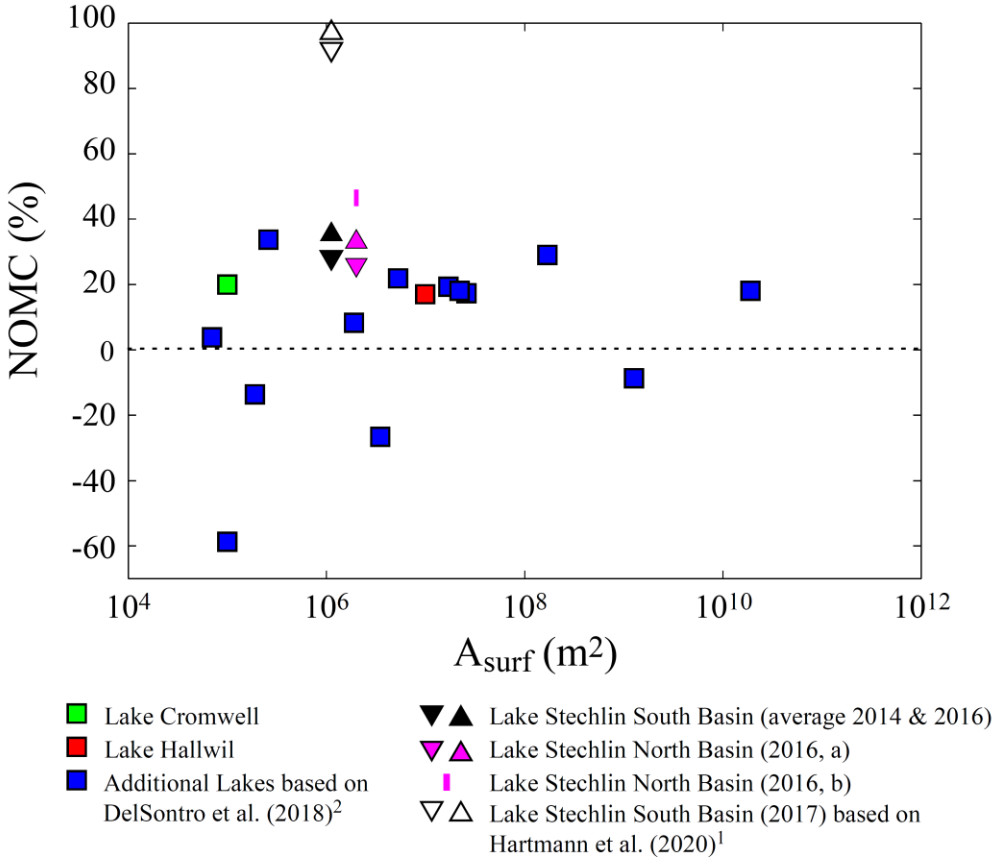

Supplementary Figure 4: The contribution of net-oxic methane production to the diffusive CH_4 emission from lakes, *NOMC*, in relation to A_{surf}.
NOMC was calculated in the different lakes from F_{surf} and F_{sed} obtained from different data sources: **Lake Hallwil** (Supplementary Table 1): F_{surf} from the "Hallwil relationship" that is based on the chamber measurements in Lake Hallwil[4]. F_{sed}, from the CH_4 pore water concentrations in the sediment core collected at 3 m water depth (F_{sed} = 2.8 mmol m^{-2} d^{-1}, Supplementary Table 1). **Lake Stechlin** (Supplementary Table 3): Lower and upper limits of F_{sed} (F_{sed} = 1.8 mmol m^{-2} d^{-1} and F_{sed} = 2.0 mmol m^{-2} d^{-1}) from the re-evaluation of the mesocosm experiments (Supplementary Table 2) providing upper and lower limit of *NOMC*, respectively (Supplementary Table 3). ***South Basin (average 2014, 2016)***: F_{surf} from the "Stechlin relationship"; ***North Basin (2016, a)*** : F_{surf} from chamber measurements; ***North Basin (2016, b)*** F_{surf} from chamber measurements combined with the , "Stechlin relationship" for the 20th of June; **Lake Stechlin *South Basin (2017)*** (Supplementary Table 4): F_{sed} derived from CH_4 pore water measured in a single sediment core by [1], considering the CH_4 gradient in the top 2 cm and at 5 cm depth, (F_{sed} = 0.08 mmol m^{-2} d^{-1} and F_{sed} = 0.26 mmol m^{-2} d^{-1}) providing upper and lower limit of *NOMC*, respectively; F_{surf} from specific wind model of [1]; **Lake Cromwell**: Data from [3]. **Additional Lakes** (Supplementary Table 5): Based on the analysis of DelSontro et al. (2018)[2].

4.2. The ratio A_{sed}/V_{SML} for the lakes investigated by DelSontro et al. 2018[2]

Analogous to Figure 4 in Günthel et al. 2019[3], Figure 1 in this study depicts *NOMC* as function of the ratio of the sediment area in the SML, A_{sed}, to the volume of the SML, V_{SML}, i.e. $R_{AV} = A_{sed}/V_{SML}$. Hence, the sediment area in the SML must be estimated for the lakes studied by DelSontro et al. 2018[2]. Note, that at least in the analysis here, the sediment area in the SML has no effect on *NOMC*, but only on the position of the *NOMC* along the x-axis.

Günthel et al. 2019[3] estimated A_{sed} by assuming a 45° slope of the lake bed. This slope is entirely unrealistic: The effective slope of the sediments providing the sediment area of the SML assuming a radially symmetric lake is 4.5° in Lake Hallwil, 4.0° in the South and 5.9° in the Northeast basin of Lake Stechlin, but not 45°. Assuming a slope of 45° of the sediments in the SML as in Günthel et al. 2019[3] substantially underestimates the correct A_{sed},, e.g. in Lake Hallwill by an order of magnitude.

Here we estimate A_{sed} and R_{AV} assuming that the lakes are radially symmetric, i.e. $r_{surf} = \sqrt{A_{surf}/\pi}$, and have a slope of 5°, alternatively of 3°, of the lake bed from the shore towards the center of the lake. The slope of 5° is motivated by the slopes in Lake Hallwil and in South and North basin of Lake Stechlin. Calculations with the slope 3° are included to illustrate the sensitivity of the position of *NOMC* along the x-axis of Figure 1 to the choice of smaller slopes (compare Figure 1 and Supplementary Figure 5). The volume of the SML is estimated as $V_{SML} = d_{SML} \cdot (A_{surf} + A_{dSML})/2$, and the sediment area as $A_{sed} = A_{surf} - A_{dSML}$, whereby d_{SML} is the surface mixed layer depth provided by DelSontro et al. 2018[2] and $A_{dSML} = \pi \cdot (r_{surf} - d_{SML}/tan\theta)^2$ is the area at the water depth of the SML. R_{AV} are provided in Supplementary Table 5.

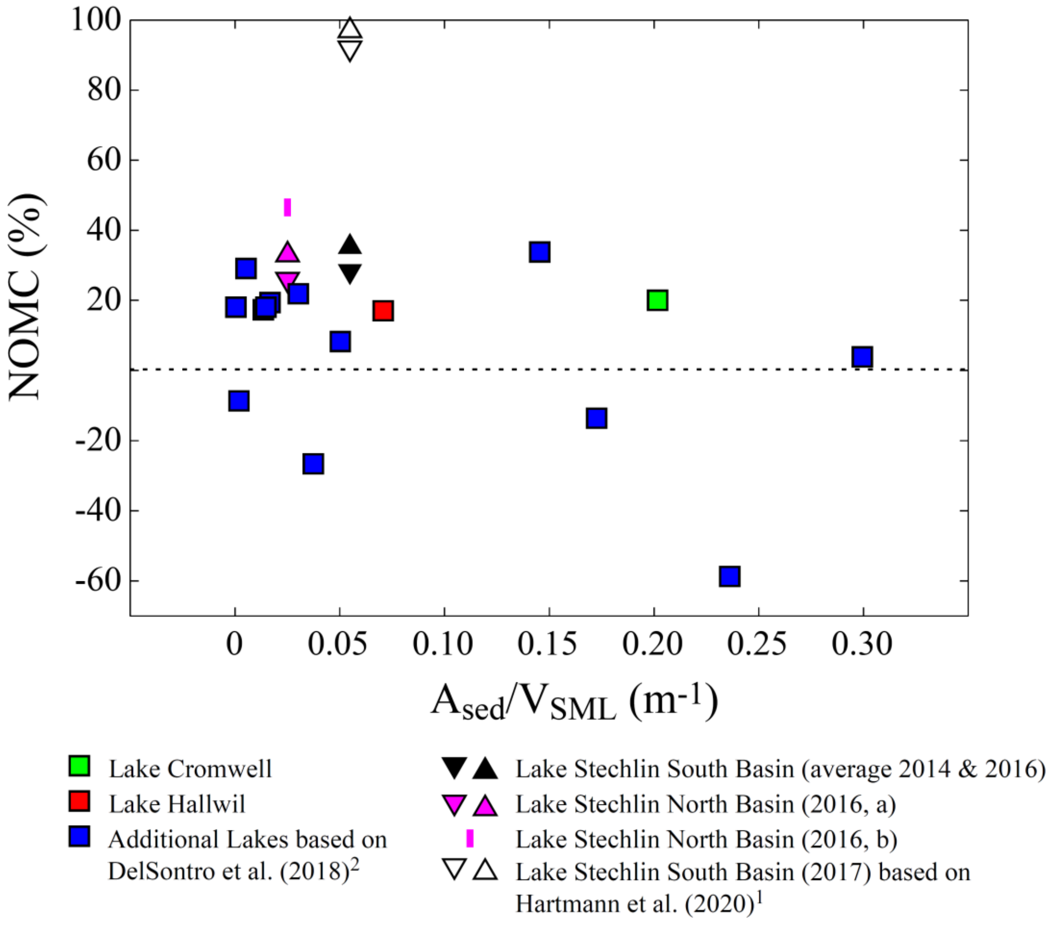

Supplementary Figure 5: The contribution of net-oxic methane production to the diffusive CH_4 emission from lakes, *NOMC*, in relation to A_{sed}/V_{SML}.
Analogous to Figure 1 but A_{sed}/V_{SML} was estimated assuming a slope angle of 3° for the lake bed. *NOMC* was calculated in the different lakes from F_{surf} and F_{sed} obtained from different data sources: **Lake Hallwil** (Supplementary Table 1): F_{surf} from the "Hallwil relationship" that is based on the chamber measurements in Lake Hallwil[4]. F_{sed}, from the CH_4 pore water concentrations in the sediment core collected at 3 m water depth (F_{sed} = 2.8 mmol m^{-2} d^{-1}, Supplementary Table 1). **Lake Stechlin** (Supplementary Table 3): Lower and upper limits of F_{sed} (F_{sed} = 1.8 mmol m^{-2} d^{-1} and F_{sed} = 2.0 mmol m^{-2} d^{-1}) from the re-evaluation of the mesocosm experiments (Supplementary Table 2) providing upper and lower limit of *NOMC*, respectively (Supplementary Table 3). ***South Basin (average 2014, 2016)***: F_{surf} from the "Stechlin relationship"; ***North Basin (2016, a)*** : F_{surf} from chamber measurements; ***North Basin (2016, b)*** F_{surf} from chamber measurements combined with the , "Stechlin relationship" for the 20th of June; **Lake Stechlin *South Basin (2017)*** (Supplementary Table 4): F_{sed} derived from CH_4 pore water measured in a single sediment core by [1], considering the CH_4 gradient in the top 2 cm and at 5 cm depth, (F_{sed} = 0.08 mmol m^{-2} d^{-1} and F_{sed} = 0.26 mmol m^{-2} d^{-1}) providing upper and lower limit of *NOMC*, respectively; F_{surf} from specific wind model of [1]; **Lake Cromwell**: Data from [3]. **Additional Lakes** (Supplementary Table 5): Based on the analysis of DelSontro et al. (2018)[2].

Supplementary References

1. Hartmann, J. F. *et al.* High spatiotemporal dynamics of methane production and emission in oxic surface water. *Environ. Sci. Technol.* **54**, 1451–1463 (2020).

2. Delsontro, T., Giorgio, P. A. & Prairie, Y. T. No Longer a Paradox : The Interaction Between Physical Transport and Biological Processes Explains the Spatial Distribution of Surface Water Methane Within and Across Lakes. *Ecosystems* **21**, 1073–1087 (2018).

3. Günthel, M. *et al.* Contribution of oxic methane production to surface methane emission in lakes and its global importance. *Nat. Commun.* **10**, 1–10 (2019).

4. Donis, D. *et al.* Full-scale evaluation of methane production under oxic conditions in a mesotrophic lake. *Nat. Commun.* **8**, 1–11 (2017).

5. Peeters, F., Fernandez, J. E. & Hofmann, H. Sediment fluxes rather than oxic methanogenesis explain diffusive CH4 emissions from lakes and reservoirs. *Sci. Rep.* **9**, (2019).

6. Yvon-Durocher, G. *et al.* Methane fluxes show consistent temperature dependence across microbial to ecosystem scales. *Nature* **507**, 488–491 (2014).

7. MacIntyre, S. *et al.* Buoyancy flux, turbulence, and the gas transfer coefficient in a stratified lake. *Geophys. Res. Lett.* **37**, 93106 (2010).

8. Lamont, J. C. & Scott, D. S. An eddy cell model of mass transfer into the surface of a turbulent liquid. *AIChe J.* **16**, 513–519

9. Lorke, A. & Peeters, F. Toward a Unified Scaling Relation for Interfacial Fluxes. *Journal of Physical Oceanography* **36**, 955–961 (2006).
